# A Dynamic Perspective on Changes in Conscientiousness, Academic Performance and the Role of Parental Academic Expectations in Chinese High School Students: A Longitudinal Study Across 2 Years

**DOI:** 10.3390/bs15060776

**Published:** 2025-06-04

**Authors:** Xing Ma, Guanjun Li, Chunquan Liu, Lei Wang

**Affiliations:** School of Psychological and Cognitive Sciences and Beijing Key Lab for Behavior and Mental Health, Peking University, Beijing 100871, China; xingm@stu.pku.edu.cn (X.M.);

**Keywords:** conscientiousness, academic engagement, academic performance, parental academic expectation, changes

## Abstract

While static conscientiousness is known to predict academic success, personality can be particularly dynamic during adolescence. This study adopted a unique change-oriented perspective to examine the longitudinal relationship between within-person changes in conscientiousness and changes in academic performance among Chinese high school students, while also exploring the moderating role of changes in parental academic expectations. Four waves of longitudinal data were collected from 453 students (265 males, *M*age = 15.42, *SD* = 0.76), with each wave spaced 6 months apart. Results indicated that the changes in conscientiousness (T2-T1) predicted the changes in academic performance (T4-T3) through the changes in academic engagement (T3-T2). However, the moderating effect of changes in parental academic expectations on the relationship between changes in conscientiousness and academic engagement was not significant. These findings go beyond static trait approaches by illustrating how dynamic changes in personality relate to evolving academic outcomes via engagement during the crucial high school years. The study highlights the importance of a dynamic perspective on personality, particularly within the developmental context of adolescence, and offers implications for interventions targeting both student traits and parental support in the Chinese educational context.

## 1. Introduction

The established link between personality traits and academic performance is well-documented, with numerous studies identifying personality as a robust predictor (Chamorro-Premuzic & Furnham, 2003; Mammadov, 2022; Noftle & Robins, 2007; Poropat, 2009; Qureshi et al., 2016). However, significantly less attention has been afforded to the dynamic interplay between changes in personality and concurrent changes in academic performance, especially during the critical developmental period of adolescence. This is a notable omission, as adolescence is characterized by substantial personality maturation, often with complex patterns of development and the lowest levels of rank-order stability (Roberts & DelVecchio, 2000; Soto & Tackett, 2015). During this stage, adolescents’ cognitive and emotional instability (Lerner & Steinberg, 2004) makes their personality traits particularly susceptible to environmental influences such as teacher perceptions, peer interactions, and crucially, the family context, including parental relationships and expectations (Caspi et al., 2005; Tackman et al., 2017), leading to significant fluctuations (Denissen et al., 2013; Roberts et al., 2006). Consequently, there is a critical gap in understanding how these dynamic personality states specifically shape high school students’ academic behaviors and contribute to their long-term outcomes. Beyond individual personality, the family environment is a key influence on adolescents’ academic development (Steinberg et al., 1992). In this context, prior research has found an association between parental academic expectations and student performance and engagement (Fan & Chen, 2001; Pinquart & Ebeling, 2020; Wilder, 2014). These expectations reflect beliefs about a child’s capabilities and future academic success. They are not passive hopes, but actively shape adolescents’ self-perceptions, motivation and behavior (Bleeker & Jacobs, 2004; Grolnick et al., 2007; Jones & Schneider, 2009; Smetana et al., 2004). High parental expectations can signal to children that their parents believe in their potential, fostering a sense of competence and encouraging greater effort. While the direct effects of static parental expectations are well-established, little is known about how changes in these expectations might interact with dynamic personality changes to influence academic outcomes over time. Therefore, this study aims to examine the relationship between intraindividual changes in personality and changes in academic performance among high school students, considering the moderating role of parental expectations.

The role of adolescents in school as students. They are expected to adhere to the prescribed student role norms formulated by the school (Albarello et al., 2018), which include investing in their studies and achieving better academic performance (Tao & Hong, 2014). Importantly, a growing body of research has found that conscientiousness, one of the Big Five personality traits, is the strongest non-cognitive predictor of academic performance. It can positively predict academic performance and academic engagement (Chamorro-Premuzic & Furnham, 2003; Furnham et al., 2013; Heaven et al., 2007; Mammadov, 2022; Noftle & Robins, 2007; Poropat, 2009; Qureshi et al., 2016; Richardson et al., 2012). Individuals with high conscientiousness are characterized by traits such as diligence, organization, responsibility, self-discipline, and a thorough approach to tasks (Poropat, 2009). Students with higher conscientiousness tend to engage in more effective study habits, exhibit greater persistence in the face of academic challenges, and are more likely to set and achieve academic goals (Chamorro-Premuzic & Furnham, 2003; Noftle & Robins, 2007). They also display higher levels of behavioral engagement and effort in their learning activities (Furnham et al., 2013; Richardson et al., 2012). Meta-analytic reviews have further supported this finding (Mammadov, 2022; Poropat, 2009), leading to a general consensus that conscientiousness facilitates behaviors conducive to learning and achievement.

However, existing research on the relationship between personality and academic performance has some limitations when it comes to examining dynamic change. First, most studies have used the cross-sectional research design, which only considers the level of the variable at one point in time for correlational analysis. Such a perspective ignores the fact that personality fluctuates significantly during this period. Focusing only on a static level overlooks the dynamic conception of personality, which acknowledges that traits can exhibit meaningful within-person change or fluctuation over time, particularly during periods like adolescence when personality is less stable (Roberts et al., 2006; Soto & Tackett, 2015). Second, although some studies have adopted a longitudinal tracking design to take personality changes into account, they analyzed the changes in variable levels at the group level over time. Such a perspective is relatively macroscopic and cannot explain the role of within-person variation (P. J. Curran & Bauer, 2011). Finally, most studies have examined the relationship between personality and future academic performance or engagement at one point in time (Dotterer & Lowe, 2011; Negru-Subtirica et al., 2019; Poropat, 2009; Qureshi et al., 2016), giving little consideration to how changes in personality during a specific period relate to changes in other predictive factors, which hinders our understanding of the dynamics between personality and academic performance during adolescence.

To gain a deeper insight into the complex nature of academic outcomes, this study adopts a dynamic perspective, specifically focusing on how changes occurring within students at different stages of their high school experience predict future changes in their academic performance. This approach, by tracking these evolving factors and their predictive utility over time, offers an integrative understanding of how different elements dynamically contribute to academic success. Such a perspective is crucial for capturing the multifaceted and evolving nature of student development and its impact on academic trajectories.

First, we propose a direct relationship between changes in conscientiousness and changes in academic performance:

**H1:**  *Changes in conscientiousness (T2-T1) are positively related to changes in academic performance (T2-T1).*

While the robust association between conscientiousness and academic performance has been well-established in cross-sectional research (Chamorro-Premuzic & Furnham, 2003; Poropat, 2009), a more nuanced understanding emerges when personality is conceptualized as a dynamic construct rather than a static trait. Adolescence represents a particularly critical period characterized by substantial personality development and considerable intraindividual variability in trait expression (Roberts & DelVecchio, 2000; Soto & Tackett, 2015). This developmental perspective is theoretically important because it recognizes that personality traits can undergo meaningful within-person changes driven by maturational processes and environmental influences (Denissen et al., 2013; Roberts et al., 2006). Empirical evidence supports the notion that adolescent conscientiousness exhibits notable fluctuations within relatively brief timeframes. For instance, longitudinal studies have documented significant intraindividual changes in conscientiousness over two-year periods during high school, highlighting the necessity of designs that capture within-person developmental trajectories (Borghuis et al., 2017; Klimstra et al., 2009; Meeus et al., 2011). These within-person changes in Big Five traits during the high school years have been systematically linked to novel experiences, evolving social role expectations, and intensifying academic demands (Lüdtke et al., 2011; Tackman et al., 2017). Such findings underscore the importance of adopting a dynamic approach to understanding the relationships between personality and academic performance during this period.

Conscientiousness has two dimensions: initiative and inhibition (Costa et al., 1991). Initiative is manifested as diligence and persistence in the face of challenges, such as struggle, inhibition is manifested as control of impulsive behavior, such as deliberation and impulse control (Costa et al., 1991; Peabody & De Raad, 2002). These facets represent core characteristics commonly associated with academic success, directly aligning with necessary student behaviors. The established static relationship between conscientiousness and academic performance can be partly understood through the selection effect perspective (Schneider, 1983). This perspective suggests that individuals are often selected for certain situations and roles based on their personality traits (Caspi et al., 2005). In this view, conscientiousness is related to academic performance, because it emphasizes diligence and persistence in tasks (Heaven et al., 2007), maintenance of order and behavior control over things beyond the regulations (Jackson et al., 2010), which are inevitable requirements for high academic performance. Thus, under this static context, conscientiousness is a positive predictor of academic performance.

We propose that changes in conscientiousness in a dynamic context can better predict subsequent changes in academic performance. This is because changes in conscientiousness likely reflect tangible alterations in the student’s engagement with academically relevant behaviors. Positive changes in conscientiousness indicate the improvement of individual initiative and inhibition. We propose that increases in conscientiousness lead to positive changes in academic performance. Students who experience greater growth in conscientiousness are likely to invest more time and energy in their academics (Barrick & Mount, 1991), adhere more closely to student rules, and exhibit a stronger pursuit of academic achievement, ultimately resulting in improved performance (Poropat, 2009). Furthermore, these students are often perceived as having greater potential and perseverance, especially when facing challenges (Duckworth et al., 2007). 

Conversely, we propose that students whose conscientiousness shows smaller increases, or even decreases, might exhibit less improvement or stability in personal initiative and inhibition, their adherence to rules, and their effortful behavior in complying with academic requirements. Under such circumstances, if smaller positive changes or decreases in conscientiousness are observed, parents and teachers could reduce their expectations of future academic performance, which in turn might affect students’ self-expectations (Simpkins et al., 2012). 

Next, we propose that changes in conscientiousness will influence subsequent changes in academic engagement:

**H2:**  *Changes in conscientiousness (T2-T1) are positively related to subsequent changes in academic engagement (T3-T2).*

This hypothesis is built on the understanding that conscientiousness is fundamentally linked to how students engage with their academic roles. Academic engagement refers to a state in which students feel energetic, have a strong sense of identity with learning, and are willing to proactively engage in their daily learning and life (Schaufeli et al., 2002). For adolescents, the student identity is primary, and its enactment involves adhering to complex and binding role norms (Albarello et al., 2018). A commonly expected student role norm is to study diligently and invest effort in academic pursuits, although not all students may fully internalize or enact this norm to the same degree. According to role theory, a crucial step in role enactment is role learning, which involves understanding the responsibilities and obligations of the role, as well as internalizing corresponding attitudes and emotions (Biddle & Thomas, 1966). We propose that conscientiousness is fundamentally linked to how students learn and enact their academic roles, thereby influencing their academic engagement. Specifically, from the perspective of orderliness, a facet of conscientiousness, schools themselves represent a structured environment focused on student safety and academic advancement. Highly conscientious students, valuing and respecting order, are more likely to align their behavior with these institutional norms, thus enhancing their academic engagement. This aligns with findings by Qureshi et al. (2016) who identified conscientiousness as a key predictor of academic engagement, attributing this to its inherent qualities of diligence and persistence (Heaven et al., 2007), supporting the link between conscientiousness and cognitive aspects of academic engagement. 

Therefore, in a dynamic context, we suggest that changes in conscientiousness can predict subsequent changes in academic engagement. An increase in conscientiousness, reflecting enhanced orderliness, discipline, proactivity (Costa et al., 1991; Roberts et al., 2014), and a more thorough internalization of student role responsibilities, would lead individuals to devote more time and effort to their studies (Trautwein et al., 2009), thereby increasing their engagement. Conversely, when the positive change in conscientiousness diminishes, students’ drive to further improve their role enactment might also lessen. This stabilization or decrease in the propensity towards diligent and orderly academic behavior (Roberts et al., 2014) could then lead to a stabilization or even a reduction in the subsequent growth of academic engagement. 

Furthermore, we propose that changes in academic engagement mediate the relationship between changes in conscientiousness and subsequent changes in academic performance:

**H3:**  *Changes in academic engagement (T3-T2) mediate the effect of changes in conscientiousness (T2-T1) on changes in academic performance (T4-T3); i.e., previous changes in conscientiousness affect subsequent changes in academic performance by influencing the current changes in academic engagement.*

The rationale for this mediation is that changes in conscientiousness are expected to translate into academic performance changes primarily through their impact on academic engagement. Conscientiousness is known to be associated with higher academic engagement and better performance (Duckworth et al., 2007; Poropat, 2009), while academic engagement itself positively influences academic performance (Dotterer & Lowe, 2011; Hagger & Hamilton, 2019; Jang et al., 2012). Our dynamic perspective proposes that changes in conscientiousness (T2-T1) influence subsequent changes in academic performance (T4-T3) primarily via changes in academic engagement (T3-T2). Specifically, increased conscientiousness can enhance organizational skills, self-discipline, and persistence (Roberts et al., 2006), leading to improved study habits, time management, and focused attention, which are characteristics of increased engagement. This increased engagement, through achieving mastery and experiencing competence, can also foster intrinsic motivation (Ryan & Deci, 2000), which in turn can further strengthen engagement.

Subsequently, this increased academic engagement reflects greater vigor, dedication, and absorption (Schaufeli et al., 2002) and manifests as more quality time on task, deeper learning strategies, and greater persistence is expected to directly support positive changes in academic performance (T4-T3). Just as these engaged behaviors foster success in static models, positive shifts in them should likewise improve performance over time. Conversely, a decrease in conscientiousness may lead to reduced academic engagement, which can also dampen intrinsic motivation (Gottfried, 1985). Such declines in engagement and motivation would then likely contribute to smaller improvements or even a decrease in subsequent academic performance.

Finally, recognizing the importance of the family context, we hypothesize a moderating role for changes in parental academic expectations:

**H4:**  *Changes in parental academic expectations (T2-T1) moderate the effect of changes in conscientiousness (T2-T1) on subsequent changes in academic engagement (T3-T2), such that the effect is strong when the range of changes in parental expectations is high and weak when the range of changes in parental expectations is low.*

Adolescents’ developmental trajectories, particularly in relation to academic aspirations, are significantly influenced by their surrounding social environment, with the family context playing a critical role (Steinberg et al., 1992). While research has extensively documented the effects of various forms of parental involvement on static academic outcomes (Hill & Tyson, 2009), there is a gap in understanding how dynamic shifts in specific parental factors, such as academic expectations, may interact with intraindividual changes in student personality traits to shape evolving academic behaviors like engagement.

Parental involvement plays an important role in influencing children’s academic outcomes (Steinberg et al., 1992; Hill & Tyson, 2009). Results of meta-analytic reviews showed that among the different forms of parental involvement, parental academic expectations had the greatest impact on children’s academic performance (Fan & Chen, 2001; Pinquart & Ebeling, 2020; Wilder, 2014). Parents set boundaries and convey expectations while trying to promote children’s health and independence. Such beliefs about competence and potential can influence children’s own beliefs through their role reorientation and future role expectations (Grolnick et al., 2007; Smetana et al., 2004), which may also affect their behavior (Bleeker & Jacobs, 2004; Jones & Schneider, 2009). 

However, existing research has primarily focused on the impact of static parental academic expectations. In contrast, our study adopts a dynamic perspective, exploring how fluctuations in parental academic expectations interact with changes in adolescents’ conscientiousness. During adolescence, perceived parental academic expectations can fluctuate (T. Curran & Hill, 2022), creating a context that can either facilitate or impede the development of personality traits such as conscientiousness and their translation into greater academic engagement. For example, when students perceive increasing parental expectations alongside their own rising conscientiousness, they may become more motivated to invest effort in their studies. Conversely, stable or declining expectations may offer less external reinforcement for such internal changes (Pinquart & Ebeling, 2020). Understanding this interplay between dynamic internal factors and contextual influences is crucial for a more comprehensive understanding of academic development.

Building on the known importance of parental academic expectations, we propose that changes in these expectations create a context that may either strengthen or weaken the positive effect of increasing conscientiousness on students’ academic engagement. For example, when parents notably raise their academic expectations, this can be a strong environmental signal. Such clear, rising expectations could improve students’ understanding and acceptance of their academic duties, particularly if these align with the student’s developing conscientiousness. In this situation, the positive link between increased conscientiousness and later academic engagement is hypothesized to be stronger. Conversely, if parental academic expectations remain stable, increase only slightly, or decrease, the environmental support or drive for translating increased conscientiousness into better academic engagement may be less pronounced.

**Figure 1:**
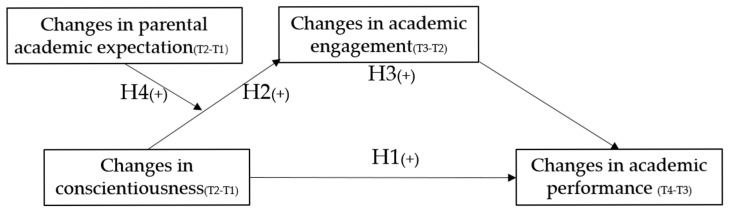
A conceptual model of the hypotheses.

The hypothesized model can now be illustrated, as shown in Figure 1.

## 2. Method

### 2.1. Participants

We conducted a two-year longitudinal study on academic health at a high school in Shandong Province, an eastern region of China characterized by moderate economic development and relatively concentrated educational resources. The first data collection (T1) was completed two weeks before the final exam of the first semester after entering high school. In the Chinese education system, students typically enter high school around the age of 15, aligning with our participants’ average age of 15.42 years (*SD* = 0.76) at the time of the first survey. We initially collected data from 539 participants in the first wave (T1). Over the two-year period, we collected data in four waves, with each wave conducted six months apart. To ensure data quality, we excluded participants who failed the attention check questions (e.g., instructions such as ‘Please select 5 for this item’), did not complete all four waves of questionnaires, or did not have academic performance records for all four waves due to factors such as student absenteeism. Our final valid sample consisted of 453 students (effective rate 84.04%). The demographics of the sample were 58.50% (265) male, 73.7% attending boarding school, and 42.50% an only child.

High school is a critical period in the physiological and psychological development of students, during which they also face considerable academic pressure and high parental academic expectations. In this context, our study focused specifically on the behavioral (e.g., academic engagement) and psychological changes of these high school students, who are navigating an often-unstable period of personality development (conscientiousness).

### 2.2. Procedure

Data collection at each time point was completed two weeks before the respective final exam of that academic period and involved two main components: self-report questionnaires and official academic records. Self-report data were collected using paper-and-pencil questionnaires. Questionnaires were administered in the classroom during designated sessions, and coordinated with the school administration. These sessions were supervised by trained research assistants from our laboratory or cooperating school staff. They provided participants with detailed instructions on how to fill out the questionnaire before participants began. The school provided us with access to the students’ official final examination grades for each relevant semester.

### 2.3. Measures

The measures of conscientiousness, academic engagement, and parental academic expectations were all administered via self-report questionnaires. A standard back-translation method was used to translate the items from English into Chinese (Brislin, 1970). All items on the scale were rated on a Likert-type response scale ranging from 1 (completely disagree/inappropriate) to 7 (completely agree/appropriate).

Conscientiousness: Conscientiousness was assessed using a scale adapted from the revised Big Five Mini-Markers (Saucier, 1994; Wei et al., 2017). This measure consisted of 8 descriptive adjectives. Participants were asked to rate the extent to which each adjective described them. Example items included “organized”, “orderly”, and “practical”. In the present study, the internal consistency for this scale, as measured by Cronbach’s alpha, was α = 0.81 at Time 1, α = 0.76 at Time 2, α = 0.78 at Time 3, and α = 0.75 at Time 4. The 6-month test-retest reliability coefficients, indicated by the correlations between scores at adjacent waves (see Table 1), were *r* = 0.27 (T1-T2, *p* < 0.01), *r* = 0.24 (T2-T3, *p* < 0.01), and *r* = 0.54 (T3-T4, *p* < 0.01).

Academic engagement: Academic engagement was measured with the student version of the Utrecht Work Engagement Scale (UWES-S; Schaufeli et al., 2002), which has been widely validated for use with student samples (Maricuțoiu & Sulea, 2019; Sulea et al., 2015). The scale comprised 17 items assessing three dimensions: Vigor (e.g., “When I’m doing my schoolwork, I feel bursting with energy”; “I feel strong and vigorous when I am studying”). Dedication (e.g., “I am enthusiastic about my studies”; “My studies inspire me”). Absorption (e.g., “When I study, I forget everything else around me”; “I feel happy when I study with seriousness and urgency”). Participants rated their agreement with each statement. The Cronbach’s alpha coefficients for the overall scale in this study were α = 0.82 at Time 1, α = 0.82 at Time 2, α = 0.85 at Time 3, and α = 0.87 at Time 4. The 6-month test-retest reliability coefficients, indicated by correlations between scores at adjacent waves, were *r* = 0.28 (T1-T2, *p* < 0.01), *r* = 0.43 (T2-T3, *p* < 0.01), and *r* = 0.44 (T3-T4, *p* < 0.01).

Parental academic expectation: Parental academic expectation was measured using 5 items adapted from an educational expectation scale (Tynkkynen et al., 2012). Participants reported on their perceptions of their parents’ expectations. Example items included “My parents expect my exam scores to always be higher than my own expectations” and “My parents expect me to enter a school with a higher ranking than my own expected entry ranking”. The Cronbach’s alpha coefficients for this measure were α = 0.77 at Time 1, α = 0.81 at Time 2, α = 0.83 at Time 3, and α = 0.80 at Time 4. The 6-month test-retest reliability coefficients, indicated by correlations between scores at adjacent waves, were *r* = 0.02 (T1-T2, *p* > 0.05), *r* = 0.39 (T2-T3, *p* < 0.01), and *r* = 0.42 (T3-T4, *p* < 0.01).

Academic performance: Academic performance was operationalized as the sum of scores obtained by each student across all academic subjects in the final examinations of each semester. In the Chinese high school context, the final exam total score is a common and objective indicator of a student’s overall academic performance for that semester.

### 2.4. Analytical Approach

Our analytical approach centered on examining change scores, rather than the levels of variables measured at single time points. This approach was adopted due to the established time-dependent nature of personality traits (e.g., conscientiousness), academic engagement, and academic performance (Wagner et al., 2019). People often perceive their life experiences in stages (Beal et al., 2005). For high school students, an academic semester naturally represents such a stage. Therefore, we focused on within-person changes occurring between consecutive semesters and quantified the magnitude of the observed variations (Grimm et al., 2012). This approach allowed us to examine how changes in variable levels within an individual from one semester to the next relate to other evolving factors, facilitating the exploration of within-person dynamic relationships.

To test our hypotheses, we calculated the changes in each variable. Multi-wave data collection allows us to examine the changes in a single variable over two time periods. In general, the amount of changes can be positive, negative, or stable. All statistical tests were performed using SPSS Statistics Version 26.0 and the PROCESS 3.5 macro (Hayes, 2013; Preacher & Hayes, 2008) was used to examine both mediation effects and moderated mediation effects. Our analyses controlled for gender, age, residence status, and whether the child was an only child. Gender and age were included because they are known to influence personality development (Caspi et al., 2005), while residence status and only child status were included as these can affect parental academic expectations via differences in contact between parents and children. To determine the bias-corrected bootstrap 95% confidence intervals (CIs), the number of bootstrap samples used in the present study was 5000. Given the potentially large size of the raw academic performance scores and to facilitate the interpretation of regression coefficients, the change scores for academic performance (e.g., performance at T4 minus performance at T3) were standardized prior to hypothesis testing. Specifically, for each relevant change interval, we calculated the mean and standard deviation of these raw change scores across all participants. Each participant’s raw change score was then converted to a z-score using the mean and standard deviation specific to that particular change interval.

## 3. Results

### 3.1. Descriptive Statistics in Four Measurements

Table 1 displays the means, standard deviations, and correlations of each variable. across four measures. The correlation coefficients of conscientiousness in four wave measures ranged from 0.16 to 0.54 (*p* < 0.01). To some extent, this highlights the changes and instability of individuals’ personalities in high school and supports the basic logic of conducting this research. Notably, academic engagement also increased over time and remained at a steady level by the end of high school, possibly due to the approaching time of the college entrance exam or the deepening of individual role learning. Interestingly, parental academic expectations were positively correlated with academic performance at T1, but negatively correlated at T2, T3, T4. A possible explanation is that parental academic expectations can have a positive impact on students when they first start school, but such expectations, if they rise over time, are likely to lead to some potentially negative consequences. In addition, the correlation coefficients for academic performance were above 0.64 for all four measures (*p* < 0.01). In terms of the entire sample, the performance has good consistency and stability in the high school stage. The previous academic foundation will help individuals to have favorable conditions in their later studies. In addition, Table 2 displays the means, standard deviations, and correlations between the study variables involved in hypothesis testing.

### 3.2. Hypothesis Testing

#### 3.2.1. Effect of Changes in Conscientiousness on Subsequent Changes in Academic Performance

Hypothesis 1 predicted that changes in conscientiousness were positively correlated with changes in academic performance. To test H1, Firstly, we first calculated the changes in consciousness (T2-T1) and academic performance (T2-T1). We then used hierarchical regression to examine the impact of changes in consciousness on academic performance and controlled for gender, age, only-child status, and whether they were resident students. With regard to Hypothesis 1, the results showed that the changes in consciousness were not significantly related to the changes in academic performance (β = 0.07, *p* > 0.05, 95% CI [−0.02 0.17]). H1 was not supported. The results suggest that personality fluctuations during students’ first year of high school may not have a direct influence on changes in academic performance over two semesters.

#### 3.2.2. The Mediation of Changes in Academic Engagement in the Relationship Between Changes in Conscientiousness and Academic Performance

For the regression model predicting changes in academic performance from both changes in conscientiousness and changes in academic engagement simultaneously, Variance Inflation Factor (VIF) tests were conducted. The VIF for changes in conscientiousness was 1.02, and the VIF for changes in academic engagement was 1.02. As all VIF values were substantially below the common threshold of 10, this indicates that multicollinearity between these predictors was not a concern in this model, satisfying this regression assumption. Furthermore, to ensure that the assumptions of the regression model were adequately met, a residual analysis was conducted. Histograms and Normal Q-Q plots of the standardized residuals indicated an approximate normal distribution. Scatterplots of standardized residuals against standardized predicted values showed no clear evidence of heteroscedasticity or non-linear patterns. The Durbin–Watson statistic for this model was 1.883, suggesting no significant concerns regarding residual autocorrelation. In Hypothesis 2 and Hypothesis 3, We hypothesize that changes in consciousness (T2-T1) are positively related to changes in academic engagement (T3-T2) and positively related to subsequent changes in academic performance (T4-T3) through changes in academic engagement. This hypothesis was tested with Model 4 of the PROCESS macro. As Table 3 shows (Model 1), changes in conscientiousness were positively associated with changes in academic engagement changes in academic performance (β = 0.18, *t* = 2.90, *p* < 0.01), which in turn was positively related to changes in academic performance (β = 0.25, *t* = 6.94, *p* < 0.001). The positive direct association between changes in conscientiousness and changes in academic performance remains significant (β = 0.17, *t* = 3.48, *p* < 0.001). Therefore, Hypotheses 2 and 3 were supported. Changes in academic engagement partially mediated the relationship between changes in conscientiousness and changes in academic performance (indirect effect = 0.05, *SE* = 0.02, 95%CI = [0.01, 0.08]). The mediation effect accounts for 21.30% of the total effect of changes in conscientiousness on changes in academic performance. 

#### 3.2.3. The Moderating Effect of Changes in Parental Academic Expectations

To check for multicollinearity among the predictors in the model predicting mediator, Variance Inflation Factors (VIFs) were calculated. The VIF for the independent variable was 1.005, for the moderator was 1.088, and for the interaction term was 1.085. All VIF values were well below the commonly accepted threshold of 10, indicating that multicollinearity was not a concern in this model. To further ensure the regression model assumptions were adequately met, a comprehensive residual analysis was performed for this moderation model. Visual inspection of histograms and Normal Q-Q plots suggested that the standardized residuals were approximately normally distributed. Moreover, scatterplots of standardized residuals versus standardized predicted values did not reveal any obvious patterns of heteroscedasticity or non-linearity. The Durbin–Watson statistic was 1.750, also indicating that residual autocorrelation was not a significant issue. H4 predicted that the effect of changes in conscientiousness (T2-T1) on changes in academic engagement (T3-T2) would be moderated by the differences in changes in parental academic expectations (T2-T1). To test the moderated mediation model, we used Model 7 of the SPSS macro PROCESS compiled by Hayes (2013). The results of the changes in parental academic expectations moderation test are shown in Table 3. As shown in Model 2 of Table 3, the predictive effect of the product (interaction term) between changes in conscientiousness and changes in parental academic expectations on changes in academic engagement was not significant (β = −0.04, *t* = −0.95, *p* > 0.05). H4 was not supported. This may reflect the highly subjective and dynamic nature of perceived parental expectations during the first year of high school, meaning shifts in these perceptions do not consistently moderate how student adjustments relate to changes in academic engagement.

## 4. Discussion

### 4.1. Summary of the Findings

In this study, we examined the relationship between changes in conscientiousness and changes in academic performance among high school students, employing a change-oriented perspective to understand adolescent personality fluctuations and academic performance. Our results showed that adolescents exhibited considerable variability in their conscientiousness traits across four measurement occasions in high school, with test–retest correlation coefficients over 6-month intervals ranging from 0.16 to 0.54. This highlights the potential instability of personality traits during adolescence (Denissen et al., 2013; Roberts et al., 2006). Furthermore, our key finding was that while initial changes in conscientiousness (T2-T1) did not show a significant direct relationship with concurrent changes in academic performance (T2-T1), they did influence subsequent changes in academic engagement (T3-T2), which in turn affected later changes in academic performance (T4-T3). However, changes in perceived parental academic expectations did not moderate the relationship between changes in conscientiousness and academic engagement. These findings highlight the crucial mediating role of changes in academic engagement in linking early changes in conscientiousness to later academic outcomes.

A noteworthy pattern emerged when comparing the non-significant direct effect of early changes in conscientiousness (T2-T1) on concurrent changes in academic performance (T2-T1) with the significant indirect effect (via changes in academic engagement T3-T2) and the significant direct effect of these same conscientiousness changes on later academic performance (T4-T3). This highlights the importance of considering temporal lags and intervening processes in understanding how personality changes translate into performance outcomes. This finding suggests that initial shifts in conscientiousness may not yield immediate, measurable changes in academic performance within the same early timeframe. Academic performance, often a cumulative outcome, likely requires a longer period for the behavioral manifestations of evolving conscientiousness (e.g., improved study habits) to fully consolidate and impact grades. 

The present study contributes importantly to the limited literature on the changes between personality and academic performance in adolescents. In contrast to the predictive power of static conscientiousness (Chamorro-Premuzic & Furnham, 2003; Furnham et al., 2013; Poropat, 2009), little attention has been paid to how the amount of change in the trait of conscientiousness affects academic performance, especially during adolescence. In this study, we chose to examine the changes in variables at different stages of high school, and the prediction of changes in one variable by changes in others helps us, to some extent, further understand the process of individual change and adaptation, particularly during the adolescent period, over time from a new perspective (Weiss & Rupp, 2011).

Furthermore, adopting a dynamic perspective, our findings reveal that the influence of conscientiousness on behavior (academic engagement) and academic outcomes persists even when considering within-person changes over time. Specifically, we highlight a key mechanism whereby initial changes in conscientiousness among high school students influence subsequent changes in academic engagement, which in turn influence subsequent changes in academic performance. This highlights the crucial mediating role of changes in academic engagement in linking early personality changes to later academic outcomes. 

We also examined the moderating role of changes in parental academic expectations, but this effect was not statistically significant. This suggests that large increases in expectations are not always beneficial (Fan & Chen, 2001; Wilder, 2014). The current findings may have emerged for a number of reasons. First, the quantitative change in parental expectations may not capture their different psychological effects (motivating for some, overwhelming for others). Second, the transition to high school often involves substantial and varied changes in students’ perceptions of parental expectations, and these changes may lead to different outcomes. Therefore, future research should not only consider whether parents continually raise expectations or maintain them at a moderate level but also aim to employ more robust measures for change in parental expectations, perhaps exploring qualitative aspects or students’ perceptions of these changes. 

Overall, the current findings are particularly relevant when attempting to understand complex student outcomes from a combinatory perspective. By investigating how conscientiousness, academic engagement, and academic performance evolve, our study offers a more comprehensive understanding of the interaction between personal traits and behavioral engagement in affecting educational outcomes. This approach responds to the call for research examining the interplay between various factors affecting academic performance, rather than considering single variables in isolation. Furthermore, our longitudinal design allows us to observe shifts in personality and engagement during adolescence and understand their impact on academic achievement over time. Such an integrative perspective is essential for developing comprehensive theoretical models to guide future research and practical interventions in educational contexts.

### 4.2. Theoretical Contributions and Practical Implications

First, this research adopts a dynamic approach to complement and offer a novel perspective from traditional static studies. Recent longitudinal research has examined the relationship between personality traits and academic achievement (Jiang et al., 2019; Negru-Subtirica et al., 2019), but such studies often focus on the relationship between levels of variables at different time points. In contrast, analyzing changes in variables provides a more nuanced understanding of individual differences in change trajectories, even among individuals who appear similar based on static levels. Therefore, the present research aims to extend the applicability of concepts often discussed in the context of static findings to the domain of within-person change over time. This addresses a gap in how static approaches fail to account for dynamic individual development over time. 

Second, by examining the relationship between changes in adolescents’ academic performance and conscientiousness, our research reveals a more nuanced pattern in the observed changes and malleability of personality during adolescence. Previous studies have often ignored the variation within individuals. In this research, we used the amount of variation as the main observation to microscopically examine the relationship between personality and academic performance. This not only inspires new ideas for future research on adolescent personality, but also has significant practical application value. Specifically, recognizing that conscientiousness is malleable during adolescence opens avenues for intervention. Educational institutions and support staff could consider implementing programs aimed at fostering conscientiousness-related skills, such as time management, organization, goal setting, and persistence, particularly during critical transition periods like the beginning of high school. Since changes in academic engagement serve as a crucial mediator, interventions could also directly target engagement by enhancing learning interest, perceived relevance of schoolwork, and providing academic support structures. Monitoring changes in both conscientiousness and engagement, rather than just static levels, might allow educators to identify students potentially at risk for declining academic performance earlier. Furthermore, our investigation into changes in parental academic expectations revealed no significant moderating effect on the relationship between changes in conscientiousness and academic engagement. This suggests that the role of parental expectations may be more complex than initially hypothesized, or that other forms of parental involvement may be more salient. 

Finally, from a change perspective, our findings suggest that the relationship between changes in conscientiousness and academic performance is mediated by academic engagement, rather than resulting from a direct effect. For example, an increase in conscientiousness may lead to greater academic engagement, which in turn improves academic performance. Following such academic gains, if engagement levels off or declines (even when conscientiousness remains relatively stable) a corresponding plateau or decrease in academic performance may occur. Therefore, for students whose grades are improving, it is important to support the continuation of academic engagement to sustain their progress. Conversely, after a decline in academic performance, which may signal reduced engagement, efforts should focus on reactivating that engagement.

### 4.3. Limitations and Future Research

A number of limitations associated with this project should be noted. Our primary analytical approach in this study involved examining changes from one stage to another. While this method was aligned with our research aim of focusing on these apparent changes, it has the limitation that observed scores inherently capture a blend of underlying developmental trends and temporary, situational fluctuations that are common during adolescence. It can be challenging to interpret these distinct sources of change using observed scores alone. Future research aiming to model these developmental dynamics with greater nuance could consider using methods such as latent change score modeling (McArdle, 2009). 

Second, the data on parental academic expectations collected in this study were based on students’ self-reports, which may potentially diverge from parents’ actual expectations (MacCann et al., 2015). This difference could introduce perceptual bias, as adolescents’ perceptions of their parents’ expectations may not accurately reflect their parents’ intentions or attitudes. Such bias may help explain the non-significant moderation effects observed in our study. Therefore, future research should include multi-informant data, such as direct reports from parents, to more accurately capture the role of parental expectations. 

Third, although students’ grades are certainly an objective indicator to measure their academic performance, they may not fully describe students’ performance during the three years of high school (Borghans et al., 2016). Future research could also use additional more enriched indicators to measure students’ academic performance, such as teachers’ evaluations.

Fourth, whether the logical relationships of absolute horizontal variables under a static state can be applied to dynamic changes still needs more research to explore and clarify. In this research, the static theory was used to derive and explain the assumptions and results in a dynamic context, which is an expansion of the existing theory. Thus, more research designs including the time dimension are needed to verify the static theory and supplement the relevant constructs.

Finally, from the perspective of reciprocal determinism (Bandura, 1978), future research also needs to consider more individual factors (e.g., emotion, motivation, and traits) and more environmental factors (e.g., school-level intervention events and teachers’ management strictness) to enrich the study of the relationship between personality and academic performance, especially considering that adolescents’ personality is highly fluctuating.

## 5. Conclusions

In sum, this study focused on examining the relationships between changes in adolescent personality traits (conscientiousness) and academic performance over the four semesters of high school, as well as the role of changes in academic engagement and parental academic expectations. Specifically, initial changes in conscientiousness (T2-T1) were found to affect subsequent changes in academic performance (T4-T3) by influencing current changes in academic engagement (T3-T2).

## Figures and Tables

**Table 1:** Means, standard deviations, and correlations of each variable in four measurements.

Variable	*M*	*SD*	1	2	3	4	5	6	7	8	9	10	11	12	13	14	15
1. C1	4.18	0.86	-														
2. C2	4.42	0.61	0.27 **	-													
3. C3	4.47	0.91	0.43 **	0.24 **	-												
4. C4	4.41	0.89	0.35 **	0.16 **	0.54 **	-											
5. AE1	4.06	1.25	0.38 **	0.14 **	0.251 *	0.26 **	-										
6. AE2	4.25	1.12	0.30 **	0.13 **	0.21 **	0.25 **	0.28 **	-									
7. AE3	4.66	1.11	0.20 **	0.22 **	0.41 **	0.28 **	0.26 **	0.4 3**	-								
8. AE4	4.51	1.19	0.26 **	0.15 **	0.33 **	0.50 **	0.35 **	0.36 **	0.44 **	-							
9. PE1	4.52	0.99	0.18 **	0.11 *	0.159 **	0.22 **	0.34 **	0.16 **	0.12 **	0.27**	-						
10. PE2	4.43	1.15	0.08	−0.11 *	0.07	0.02	0.08	0.06	0.02	0.05	0.02	-					
11. PE3	4.11	1.26	0.08	0.06	−0.10 *	0.01	0.00	0.01	0.08	0.06	0.02	0.39 **	-				
12. PE4	4.13	1.29	0.01	0.02	0.01	0.08	0.10 *	0.02	0.12 **	0.17 **	0.03	0.26 **	0.42 **	-			
13. AP1	503.28	72.48	0.26 **	0.10 *	0.20 **	0.23 **	0.14 **	0.23 **	0.15 **	0.15 **	0.09	−0.17 **	−0.24 **	−0.11 *	-		
14. AP2	542.89	65.37	0.26 **	0.17 **	0.19 **	0.24 **	0.170 **	0.26 **	0.16 **	0.17 **	0.09 *	−0.19 **	−0.21 **	−0.14 **	0.79 **	-	
15. AP3	529.37	63.97	0.30 **	0.16 **	0.21 **	0.24 **	0.169 **	0.24 **	0.17 **	0.18 **	0.12 **	−0.21 **	−0.22 **	−0.11 *	0.85 **	0.89 **	-
16. AP4	560.44	72.72	0.19 **	0.19 **	0.16 **	0.19 **	0.12 *	0.11 *	0.22 **	0.15 **	0.13 **	−0.18 **	−0.16 **	−0.10 *	0.64 **	0.74 **	0.86 **

*N* = 453. C1 = Conscientiousness T1, AE1 = Academic engagement T1, PE1 = Parental academic expectations T1, AP1 = Academic performance T1. * *p* < 0.05. ** *p* < 0.01.

**Table 2:** Means, standard deviations, and correlations of the variables.

Variable	*M*	*SD*	1	2	3	4	5	6	7
1. Gender	-	-	-						
2. Age	15.42	0.76	−0.01	-					
3. Residential	-	-	0.03	−0.13 **	-				
4. Only Child	-	-	0.25 **	0.13 **	−0.36 **	-			
5. Conscientiousness (T2-T1)	0.24	0.91	0.20 **	0.04	−0.02	0.08	-		
6. Academic engagement (T3-T2)	0.40	1.19	0.04	−0.04	0.08	0.05	0.14 **	-	
7. Academic performance (T4-T3)	31.07	37.38	0.16 **	0.13 **	0.12 *	−0.02	0.22 **	0.33 **	-
8. Parental academic expectations (T2-T1)	−0.09	1.51	0.02	−0.03	−0.09	0.11 *	0.06	0.06	−0.02

*N* = 453. Gender: 1 = male, 2 = female. Residential: 1 = yes, 2 = no. Only child at home: 1 = yes, 2 = no. * *p* < 0.05. ** *p* < 0.01.

**Table 3:** Testing the mediation effect and the moderated mediation effect.

Predictors		Model 1 AE (T3-T2)			AP (T4-T3)		Model 2 AE (T3-T2)		
	β	*SE*	*t*	β	*SE*	*t*	β	*SE*	*t*
1. Gender	−0.02	0.12	−0.19	0.26	0.09	2.81 **	−0.01	0.12	−0.10
2. Age	−0.06	0.07	−0.83	0.20	0.06	3.56 ***	−0.05	0.07	−0.73
3. Residential	0.27	0.14	2.04 *	0.21	0.11	2.03 *	0.29	0.14	2.11 *
4. Only Child	0.20	0.12	1.58	−0.13	0.10	−1.32	0.19	0.13	1.48
5. C (T2-T1)	0.18	0.06	2.90 **	0.17	0.05	3.48 ***	0.18	0.06	2.83 **
6. AE (T3-T2)				0.25	0.04	6.94 ***			
7. PAE (T2-T1)							0.04	0.01	1.08
8. C × PAE							−0.04	0.01	−0.95
*R* ^2^		0.03			0.18			0.04	
*F*		2.95 *			16.84 ***			2.39 *	

*N* = 453. Gender: 1 = male, 2 = female. Residential: 1 = yes, 2 = no. Only child at home: 1 = yes, 2 = no. C (T2-T1) = Changes in conscientiousness. AE (T3-T2) = Changes in academic engagement. AP (T4-T3) = Changes in academic performance. PAE (T2-T1) = Changes in parental academic expectations. * *p* < 0.05. ** *p* < 0.01. *** *p* < 0.001.

## Data Availability

Data are available from the corresponding author upon reasonable request.
